# Hydrogen Production by Methanol Steam Reforming on Copper Boosted by Zinc–Assisted Water Activation**

**DOI:** 10.1002/anie.201106591

**Published:** 2012-02-15

**Authors:** Christoph Rameshan, Werner Stadlmayr, Simon Penner, Harald Lorenz, Norbert Memmel, Michael Hävecker, Raoul Blume, Detre Teschner, Tulio Rocha, Dmitry Zemlyanov, Axel Knop-Gericke, Robert Schlögl, Bernhard Klötzer

**Affiliations:** Institute of Physical Chemistry, University of InnsbruckInnrain 52A, 6020 Innsbruck (Austria); Birck Nanotechnology Center, Purdue University1205 West State Street, West Lafayette, IN 47907-2057 (USA); Department of Inorganic Chemistry, Fritz Haber Institute of the Max Planck SocietyFaradayweg 4-6, 14195 Berlin (Germany)

**Keywords:** copper/zinc alloys, methanol steam reforming, surface chemistry, water activation, X-ray photoelectron spectroscopy

For use of polymer electrolyte membrane fuel cells (PEMFC) in mobile power applications, an efficient source of CO-depleted hydrogen is needed. To avoid technical and safety problems of hydrogen handling, storage, and transport, methanol can be used as practical and abundant energy carrier for on-board H_2_ generation, as it has the advantage of a high energy density. Hydrogen generation from methanol can be performed by catalytic methanol steam reforming (MSR): CH_3_OH+H_2_O→CO_2_+3 H_2_. Methanol conversion must be carried out with very high CO_2_/H_2_ selectivity to avoid CO poisoning of the fuel-cell anode. A number of promising selective MSR catalysts are already available. Apart from advanced copper-based catalysts,^1^, ^2^ special attention is presently paid to the highly MSR-selective reduced state of Pd/ZnO,^3^ containing a particularily stable intermetallic PdZn (1:1) active phase.^3^, ^4^ Therefore, we recently studied related “inverse” near-surface PdZn intermetallic phases, showing that three-dimensional PdZn active site ensembles are equally important for selective dehydrogenation of methanol (thus avoiding CO) and for efficient water activation.^5^ For the less costly Cu/ZnO catalysts, originally designed for methanol synthesis, improvements towards a technical MSR application regarding sintering stability, pyrophoricity, and selectivity are still required. Empirical development of Cu/ZnO catalyst preparation and activation has aimed in a particularily large Cu^0^–ZnO contact.^6^ Nevertheless, it is very difficult to derive an unambiguous causality for the role of the contact on technical catalysts. It is known that zinc leads to an improvement in the desired properties, but a clear assignment of a predominant promotional effect (both from the theoretical and experimental side) is still missing. In the Cu/ZnO literature, seemingly incompatible model interpretations can be found, involving the “metallic copper model”,^7^ the “special site model”,^8^ the “morphology model”,^7^, ^9^ the “spillover model”,^10^ and last but not least the “Cu-Zn alloy model”.^8^, ^11^ Consequently, the Cu-ZnO(H) contact most likely constitutes a combination of promotional effects. The central aim of our study is to highlight the aspect of zinc-promoted water activation. This is achieved by using an ultrahigh-vacuum (UHV) “inverse” model catalyst approach, which, in contrast to investigations on real catalyst systems, allows the zinc segregation behavior and the changes in redox chemistry of both copper and zinc to be better followed. This provides a solid basis for directional promotion of microkinetic steps leading to enhanced CO_2_ selectivity.

A series of CuZn near-surface alloy and bulk brass samples were tested. The near-surface alloy preparations on Cu foil involved variable thermal Zn evaporation and thermal annealing conditions. Commercial bulk brass foil samples with 10 to 37 weight % Zn content were cleaned by usual UHV procedures. The respective bimetallic pre-reaction state was characterized by depth-resolved X-ray photoelectron spectroscopy (XPS) and Auger electron spectroscopy (AES) analysis of the alloy composition. Quantitative kinetic MSR reaction studies were then performed in a UHV-compatible all-glass reaction cell operating between 10^−10^ and 1000 mbar. Finally, simultaneous “in situ” analysis using product detection by mass spectrometry and ambient-pressure XPS (AP-XPS) was performed at the HZB/BESSY II synchrotron (for details see the experimental section in the Supporting Information). On this basis, we are able to show that the CO_2_ selectivity and activity in MSR strongly scales with the available Cu(Zn)^0^/Zn(ox) interface, which forms in situ by partial oxidative Zn segregation in the MSR gas phase on a suitable near-surface alloy “pre-catalyst”. It should be emphasized that the exact degree of initial Zn doping is crucial to establish a maximum Cu(Zn)^0^/Zn(ox) interface and thus activity. Water activation and total oxidation toward CO_2_ is inhibited on bulk and surface-clean, thermally annealed, structurally equilibrated copper foil, which is a particularily unreactive state of Cu (for a brief discussion of potential Cu activators other than Zn, see the Supporting Information, S5). Near-surface alloys that were too Zn-rich and all investigated bulk brass samples also showed complete deactivation by Zn(ox) surface passivation. By systematic variation of the Zn amount and of the annealing temperature, the most active and selective CuZn near-surface alloy preparation was empirically identified: evaporation of 5 to 12 monolayers (ML) of Zn onto Cu foil at 300 K, followed by thermal annealing in vacuum at 523 K for 10 min.

To characterize the optimized bimetallic “pre-catalyst”, 5 or 12 ML Zn were deposited under UHV conditions onto clean Cu foil at about 300 K, followed by stepwise thermal annealing up to 653 K. The corresponding AES effects are shown in Figure 1 a. Two stability plateaus are discernible. It is evident that annealing at about 400 K gives rise to a circa four-times higher Zn concentration within the first few layers of the sample than annealing at 500 K and above.

**Figure 1:**
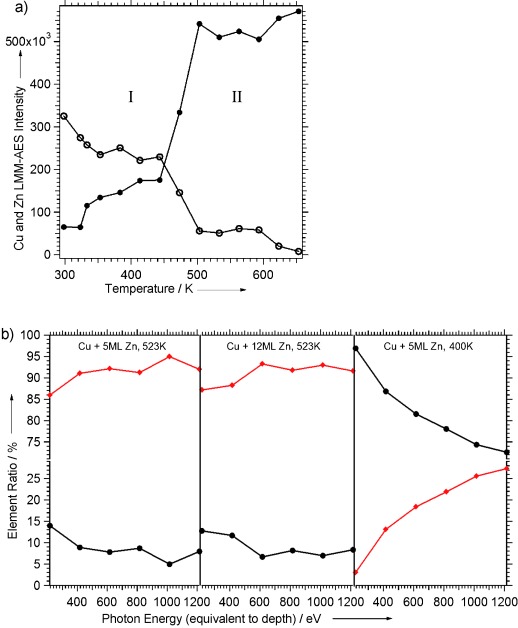
a) Peak-to-peak intensities of the differentiated Zn-L_3_M_45_M_45_ and Cu-L_3_M_45_M_45_ Auger peaks as function of annealing temperature. Initially 12 ML of Zn were deposited at about 300 K on clean Cu foil and annealed for 5 min at the respective temperature. ○: Zn-L_3_M_45_M_45_(1G), •: Cu-L_3_M_45_M_45_(1G). b) Element ratio of Cu and Zn [%] as a function of the analysis depth, derived from the Cu 3p (♦) and Zn 3p (•) XPS signals (depth profile by photon energy variation from 215 to 1215 eV, corresponding to about 0.5–1.3 nm IMFP (inelastic mean free path) of photoelectrons). Preparation conditions: evaporation of 5 or 12 ML of Zn at about 300 K followed by thermal annealing at 400 K or 523 K.

The approximate Cu:Zn=9:1 intensity ratio after annealing at 500 K and beyond can be used to estimate the mean Zn content of the AES-visible near-surface regions (kinetic energies of LMM-Auger transitions: 920 eV (Cu) and 994 eV (Zn), IMFP (inelastic mean free path) ca. 1.4 nm (Cu) and ca. 1.6 nm (Zn) at 900–1000 eV^12^). Assuming an extremely “flat” Zn-in-Cu concentration gradient for 523 K annealing, which is verified by the XPS depth profiling data of Figure 1 b, quantification of the AES results can be attempted without considering variable intensity contribution with increasing probe depth. Moreover, excitation for the AES transitions was provided using MgK_α_ radiation, thus as a reasonable approximation for the relative Auger sensitivities the respective Cu 2p and Zn 2p XPS cross-sections can be used. On this basis a Cu:Zn ratio of about 10:1 was estimated for the relatively narrow surface region detected by AES. As 12 ML of Zn at this ratio corresponds to a thickness of a 10:1 alloy region on top of the deeper Cu bulk of about 31 nm (ca. 12×10 layers ×0.26 nm layer thickness), any concentration gradient within the first few layers and any gradient-dependent intensity attenuation can be neglected. The same estimation, applied to the 400 K–450 K annealed state, results in a circa 45:55 Cu:Zn ratio, suggesting the presence of a β-brass layer (region I in Figure 1 a) becoming thermally unstable above 450 K. In the range 450 K–500 K the transition from near-surface β-brass to α-brass (region II in Figure 1 a) occurs, and finally a thermal stability range of the near-surface α-brass state between 500 K and 600 K can be deduced from Figure 1. Naturally, owing to the presence of the almost infinite Cu bulk as nearby diffusional “sink”, the stability ranges of the near-surface alloys are strongly shifted to low temperatures in comparison to their bulk counterparts.

To back up the AES quantification of the Zn content and gradient within the near-surface region, variation of the XPS probe depth by photon energy variation was performed. Figure 1 b shows the large differences in the resulting Zn near-surface distribution between annealing of 5 ML Zn below about 450 K and above 503 K, followed by contact to the ambient at about 298 K. For annealing at 523 K, the Cu:Zn≍10:1 estimation derived from Figure 1 a is complemented by a Cu:Zn≍12:1 estimation for deeper layers of the 5 and 12 ML preparations, and only the outermost region showed minor oxidative Zn enrichment.

The MSR activity/selectivity of the CuZn≍10:1 alloy was quantitatively studied in comparison to clean Cu by temperature-programmed reaction in an UHV-compatible high-pressure cell operated as a recirculating batch reactor under realistic reaction conditions. Figure 2 (upper panel) reveals that the CuZn≍10:1 surface alloy quickly converts CH_3_OH and water to CO_2_ and formaldehyde (HCHO) with virtually no CO formation in the entire temperature region between 530 K and 623 K, which is the upper limit in the batch reactor tests. In contrast, on clean Cu hardly any conversion of methanol is observed.

**Figure 2:**
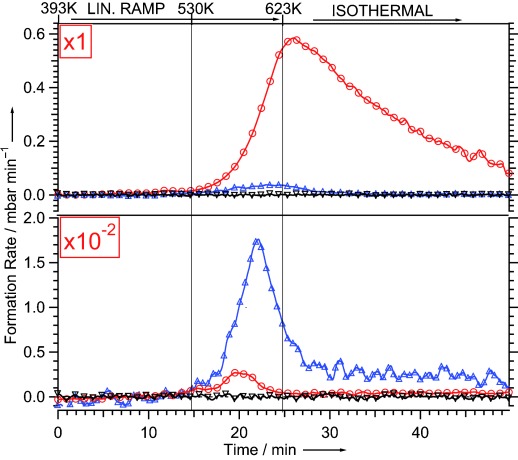
Temperature-programmed MSR on the CuZn≍10:1 near-surface alloy (upper panel) versus clean Cu foil (lower panel). Reaction conditions: 12 mbar methanol, 24 mbar water, 977 mbar He; linear temperature ramp (9.0 K min^−1^) up to 623 K, followed by isothermal reaction for 25 min. The decrease of the CO_2_ formation rate in the isothermal region is caused by progressive reactant consumption (methanol conversion after 50 min: ca. 85 %). ○ CO_2_, ▿ CO, ▵ HCHO.

An activation energy of about 93 kJ mol^−1^ for CO_2_ formation was estimated from the initial rate increase (see the Supporting Information, Figure S1). At about 600 K, HCHO is formed on both catalysts to some extent, but a high activity/selectivity toward CO_2_ is only present on CuZn≍10:1. Clean Cu only catalyzes the selective dehydrogenation of methanol to formaldehyde. Therefore the balance of dehydrogenation vs. subsequent total oxidation by water governs the HCHO:CO_2_ product ratio. A strong kinetic inhibition of water activation on clean, unactivated Cu can thus explain the huge difference to the CO_2_-active CuZn system.

The MSR-induced segregation behavior of initial CuZn≍10:1 at ambient reactant pressure between 300 K and 543 K is illustrated in Figure 3 by the Zn 3d and Cu 3d valence-band XPS spectra acquired in situ. Starting at 300 K from the bimetallic state (Figure 3 a), heating to 453 K already induces a Zn 3d shift toward higher binding energy (BE) and a pronounced relative increase of Zn 3d versus Cu 3d intensity, indicating the onset of oxidative Zn segregation to the surface. Up to 543 K, the gradual transition from the bimetallic state to a mixed Zn(bimetal)–Zn(ox) state further proceeds, as can be deduced from the respective Zn 3d peak deconvolution in Figure 3 b showing approximately equal contributions of bimetallic and oxidized Zn around 550 K (for details of the deconvolution procedure, see the Supporting Information, S2). The fact that the Zn3d/Cu3d atom ratio also increases from about 0.13 at 300 K up to a value of about 0.36 at 543 K (detailed temperature dependence of Zn/Cu ratio shown in the Supporting Information, Figure S2 a), is interpreted in terms of optimized surface wetting of Cu(Zn)^0^ by “interfacial Zn(ox)”, rather than purely three-dimensional growth of bulk ZnO islands, which would be much less sensitively detected at *hν*=130 eV. The roughly equal contributions of oxidized and bimetallic Zn3d components, in combination with the pronounced Zn:Cu intensity maximum at 543 K, suggest that the evolving active state is characterized by the presence of an only partially Cu(Zn)^0^-blocking Zn(ox) layer with a maximum number of Cu(Zn)^0^-Zn(ox) interfacial active sites. At 543 K, also the onset of CO_2_ formation on initial CuZn≍10:1 is evident from Figure 2 (upper panel).

**Figure 3:**
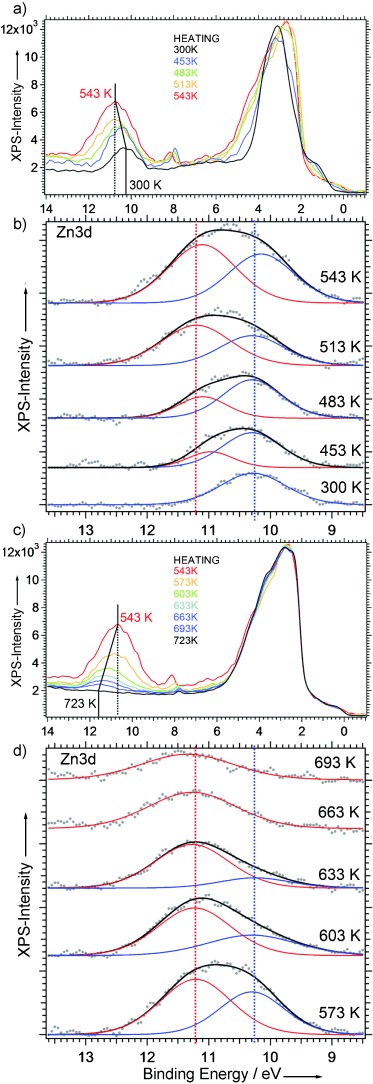
a, c) AP-XPS spectra (Zn 3d and Cu 3d regions) recorded at *hν*=130 eV in situ during MSR (0.12 mbar methanol+0.24 mbar water) starting from CuZn≍10:1. b, d) Corresponding Zn 3d peak deconvolution; red: BE≍11.2 eV, Zn(ox); blue: BE≍10.25 eV, bimetallic ZnCu. Details of Zn 3d binding energy and intensity trends are given in the Supporting Information, S2.

Nevertheless, the exact spatial distribution of “interfacial Zn(ox)” (local ensembles consisting of a few atoms vs. extended, more or less flat islands) and the predominant Zn-coordination chemistry within (distribution of Zn-Cu^0^, Zn-O and Zn-OH bonds) remains to be clarified.

At temperatures above 543 K, progressive Zn (intensity) loss is obvious from Figure 3 c,d, whereas the Zn 3d BE maximum position still shifts further up to about 11. 5 eV. Complete loss of the bimetallic Zn component is evident at >633 K in Figure 3 d, whereas total loss of all Zn is evident from the 723 K spectrum in Figure 3 c. As the pathways to reduce surface Zn intensity are three-dimensional clustering of Zn(ox) and/or reduction of Zn(ox) to Zn^0^, either followed by diffusion into the deep bulk or by desorption, at present we can only suggest a combination of these processes. However, Zn intensity loss relative to Cu^0^ necessarily means loss of active Cu^0^/Zn(ox) interface, both if three-dimensional clustering or reductive Zn loss dominates.

We strongly emphasize the absence of detectable amounts of oxidized Cu species under reaction conditions, as evident from both the core-level and valence-band Cu regions (see for example, the largely invariant “metallic” Cu 3d region in Figure 3 a,c).

The predominant oxidation of Zn is also supported by the O 1s intensity trend, which closely tracks the behavior of the Zn(ox) intensity (for details, see the Supporting Information, S4). Moreover, a continous O 1s trend to higher BE from 531.2 eV to about 533 eV between 453 K and 723 K is interpreted in terms of an increasing contribution of hydroxylated surface species (ZnO ca. 531.6 eV vs. Zn(OH) ca. 533.5 eV,^13^ Cu–OH ca. 531.9 eV, and adsorbed water ca. 533.0 eV^14^). A gradual change of the Cu(Zn)^0^/Zn(ox) active state to a less-active Zn-depleted, but partially hydroxylated, Cu^0^ state above 633 K is also inferred from the less-than-exponential temperature dependence of the reforming activity above 633 K. As total Zn loss is evident at about 700 K (Figure 3 c), the residual reforming rate, combined with a BE as high as 533 eV for O 1s, is attributed to the now activated Cu foil covered by a rather small amount of reactive OH groups (for details, see the Supporting Information, S5).

In conclusion, the Cu:Zn≍10:1 “pre-catalyst” state provides an appropriate near-surface Zn loading for MSR-induced segregation to yield submonolayer Zn(ox) coverages and therefore a high abundance of bimetal-Cu(Zn)^0^/Zn(ox) interface at about 550 K, as depicted in Scheme 1. This, in turn, creates optimized conditions for bifunctional catalyst operation: Cu(Zn)^0^ regions favor selective methanol dehydrogenation to formaldehyde, whereas redox-active Cu(Zn)^0^-Zn(ox) sites^8^, ^15^ assist in water activation and the transfer of hydroxide or oxygen to the latter, thus providing optimum conditions for high CO_2_ activity and selectivity.

**Scheme 1:**
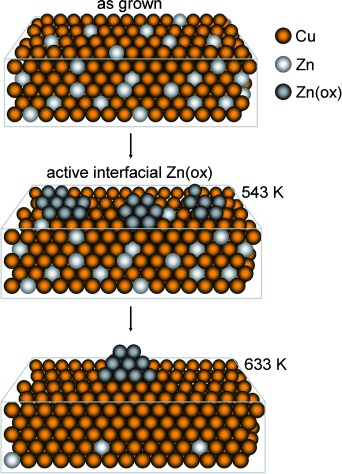
Upper panel: pre-catalytic “as-grown” CuZn≍10:1 bimetallic state. Middle panel: superior “in situ” active surface state with maximum wetting of Cu(Zn)^0^ by interfacial Zn(ox). Lower panel: High-temperature state after major Zn intensity loss by three-dimensional Zn(ox) clustering and/or bulk diffusion/desorption of Zn^0^.

On clean and unactivated Cu foil, dehydrogenation ceases with formaldehyde, but also fully Cu^0^-blocking Zn(ox) coverages cause complete deactivation.

As a perspective for future research, the detailed reaction mechanism of CO_2_ formation from water-derived O or OH species and formaldehyde remains to be clarified. So far, intermediates originally predicted for the metallic Cu^0^ surface, such as dioxomethylene^16^, ^17^ and hydroxymethoxy, which was recently proposed in between formic acid and formaldehyde,^18^ remain to be verified or disproved. Consequently it is important to study not only bimetallic Cu(Zn)^0^ sites, but also interfacial Cu(Zn)^0^/Zn(ox) and Zn(ox) surface sites. Finally, the role of reactive formate intermediates, again on (bi)metallic, oxidic and interfacial sites, should be studied in more detail. Promotion of formate reactivity has already been reported both for Zn-modified^8^ and O_ads_-modified Cu(111).^19^ The high specific methanol synthesis activity of Cu/ZnO catalysts was previously ascribed to α-brass formation.^20^ In this context, “bridging” formate species located at special Cu-Zn sites were reported.^21^

As the main advantage of our model system is both a high abundance and a close spatial vicinity of bifunctional active surface regions, we emphasize possible implications for selectivity tuning of Cu-Zn-based “real” MSR catalysts. Based on controlled Zn(ox) segregation from sufficiently diluted bimetallic Zn-in-Cu precursors to yield a similar Cu(Zn)^0^–Zn(ox) surface microstructure, the directional promotion of formaldehyde total oxidation, combined with enhanced decarboxylation selectivity of Cu-Zn-bonded formate species to suppress CO, appears feasible.

## Notes

Detailed facts of importance to specialist readers are published as ”Supporting Information”. Such documents are peer-reviewed, but not copy-edited or typeset. They are made available as submitted by the authors.
