# Supplementary material for: Hydrogen Production by Methanol Steam Reforming on Copper Boosted by Zinc–Assisted Water Activation
Source: Angew Chem Int Ed Engl. 2012 Feb 15;51(12):3002–6. doi: 10.1002/anie.201106591 (PMC3556650; doi:10.1002/anie.201106591)
Supplement: Supplementary file 1 [file anie0051-3002-SD1.pdf]

Angewandte Chemie
Eine Zeitschrift der Gesellschaft Deutscher Chemiker

Supporting Information

© Wiley-VCH 2012

69451 Weinheim, Germany

# Hydrogen Production by Methanol Steam Reforming on Copper Boosted by Zinc-Assisted Water Activation **

*Christoph Rameshan, Werner Stadlmayr, Simon Penner, Harald Lorenz, Norbert Memmel, Michael Hävecker, Raoul Blume, Detre Teschner, Tulio Rocha, Dmitry Zemlyanov, Axel Knop-Gericke, Robert Schlögl, and Bernhard Klötzer**

anie_201106591_sm_miscellaneous_information.pdf

**Experimental Section**

As reference catalyst and substrate for CuZn near-surface alloy formation, ultra-clean copper foil (Goodfellow, purity 99.999%, 0.10 mm thick, size 3.5 $cm^2$) was used without further activation procedures. Surface preparation and characterization involved the usual cycles of Ar sputtering and thermal annealing (6.0 x $10^{-5}$ mbar Ar, 2 keV, 1 µA sample current; T = 973 K), till XPS and AES spectra without evidence for impurity traces were obtained. For near-surface alloy preparation, we evaporated 5 or 12 ML of Zn metal onto the clean Cu surface, followed by thermal annealing at temperatures between 300 K and 653 K. Brass reference samples with Zn contents of 10, 15 and 37% were derived from Goodfellow (CuZn37) and Schlenk Metallfolien (CuZn10 and CuZn15).

Measurements were performed using two different experimental setups:

*Setup 1: Surface preparation and -analysis chamber with attached high-pressure batch reaction cell*

The UHV system with attached all-glass recirculating batch reactor, described in [1], is designed for quantitative catalytic/kinetic studies up to 1 bar on polycrystalline foils, detecting products by online MS analysis (HP GC-MS System G1800A) via a capillary leak and/or by conventional GC-MS analysis via column injection. MS signals of methanol, $CO_2$, CO, $H_2$ and $CH_2O$ were externally calibrated and corrected for fragmentation. On this basis also stoichiometric reforming of methanol toward $CO_2$:$H_2$ = 1:3 on Cu:Zn~10:1 could be verified. Ex-situ surface analysis was performed using a XPS/AES/LEIS spectrometer (Thermo Electron Alpha 110) and a twin Mg/Al anode X-ray gun (XR 50, SPECS). Zn deposition using a home-built Knudsen cell filled with Zn shot (Sigma-Aldrich, 99.99%) was controlled by a quartz-crystal microbalance. All MSR reactions were conducted with degassed methanol/water mixtures of a 1:10 composition of the liquid phase, corresponding to a partial pressure ratio of 1:2 = methanol:water at ~300 K. The reactor was heated at a constant linear rate of around 9 K/min to the final, maximum accessible temperature of 623 K and then kept isothermally at this temperature for ~25 min. From the product partial pressures vs. time plots the reaction rates were obtained by differentiation and are given in partial pressure change per minute [mbar/min].

*Setup 2: Ambient pressure (AP)-XPS spectrometer for in-situ catalyst characterization*

The vacuum chamber operated at HZB/BESSY II (at beamline ISISS-PGM) allowed us to perform *in-situ* photoelectron spectroscopy up to 1 mbar total reactant pressures [2]. The Cu foil or CuZn samples were positioned inside the high-pressure analysis chamber ~2 mm away from a 1 mm aperture, which is the entrance to the differentially-pumped electrostatic lens

system separating gas molecules from photoelectrons focused toward the SPECS hemispherical analyzer. Binding energies were referred to the Fermi edge recorded after each scan.

The element ratio of Cu and Zn (in %) was derived as a function of the analysis depth from the Cu3p and Zn3p XPS signals, whereby photon energy can be converted into kinetic energy by subtraction of ~90 eV for Zn3p and ~75 eV for Cu3p. Cross section correction of the Cu:Zn ratio was based on data from: http://ulisse.elettra.trieste.it/services/elements/WebElements.html. Because the Cu3p and Zn3p regions were recorded with the same photon energy in one step, correction of mirror adsorption phenomena and photon flux was not necessary.

The temperature was measured by a K-type Ni/NiCr thermocouple spot-welded onto the side of the sample and temperature-programmed heating was done by an IR laser from the rear. Sample cleaning procedures consisted of repeated cycles of $Ar^+$ sputtering at room and elevated temperatures, annealing up to 950 K in UHV, and exposure to $O_2$, followed by flashing to 950 K for 60 s in UHV.

The CuZn near-surface alloy samples were prepared in setup 1 and then transferred to the HZB/BESSY II. As they were exposed to ambient conditions in between preparation and spectroscopic analysis, signs of oxidative Zn segregation were visible, especially at low photoelectron kinetic energies. On the low-temperature (≤ 400 K) annealed samples, oxidative Zn segregation was observed to be strong, whereas the 523 K annealed samples turned out to be stable under ambient conditions, i.e. the outermost region showed negligible oxidative Zn-enrichment.

The AP-XPS characterization of the model catalyst surface under methanol steam reforming conditions was complemented by simultaneous quadrupole mass-spectrometry (QMS) analysis of product/reactant partial pressure changes. In contrast to the considerably more sensitive and quantitative rate measurements in the batch reactor cell of setup 1, the analysis of compositional changes of the gas phase at HZB/BESSY II was performed in the flow reactor mode and was experimentally accessible also in the temperature range above 623 K.

## Supplemental Results

**S1:** Estimation of activation energy for $CO_2$ formation on initial CuZn~10:1 in the recirculating batch reactor of setup 1, based on the $CO_2$ rate data of Figure 2 (main text). To minimize rate changes by changes of the reactant/product pressures, analysis was limited to the initial rate increase between 530K and 550K, where hardly any reactant consumption/product formation takes place.

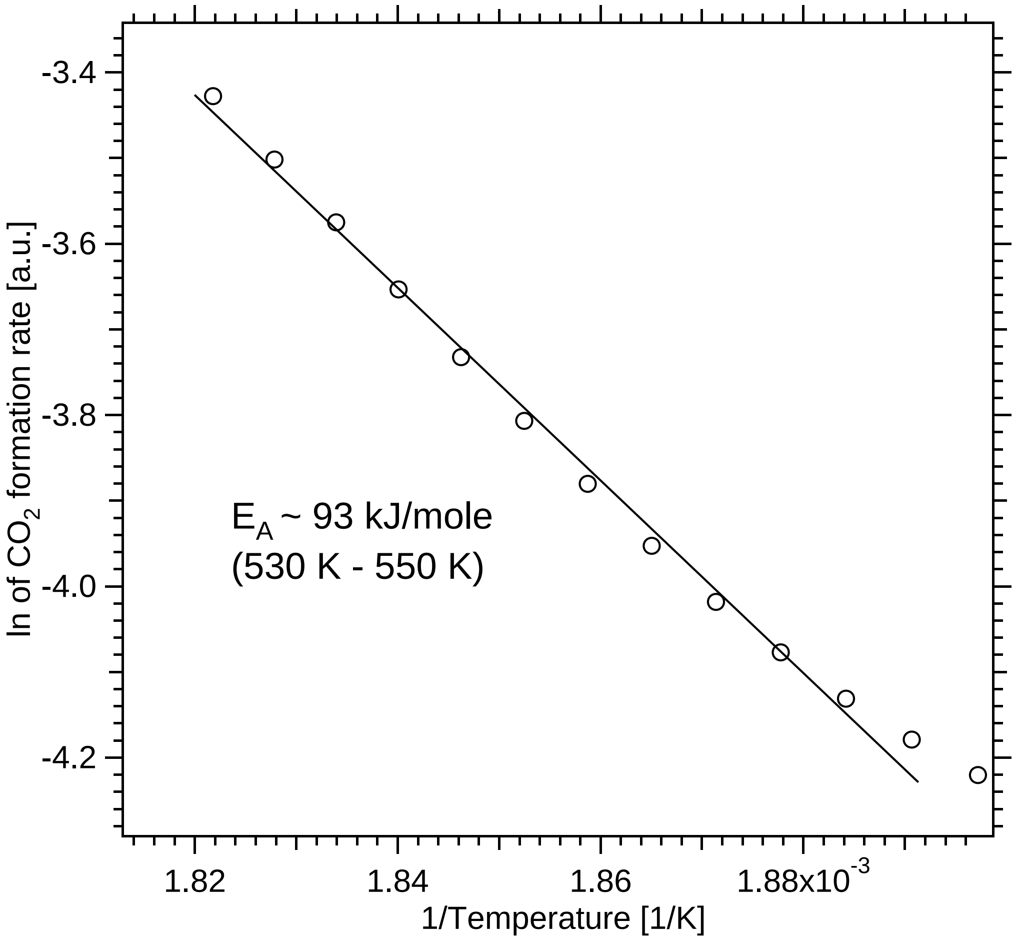


Fig. S1: Arrhenius analysis of recirculating batch reactor $CO_2$ rate data obtained in setup 1 between 530 K and 550 K (compare Figure 2, upper panel, in the main paper).

**S2:** Supporting information to Figure 3 (main text) is shown in Fig. S2: (a) Quantification of mean Zn:Cu surface ratio derived from the Cu3d/Zn3d intensity ratio. (b) Change of binding energy at the Zn3d peak maximum with reaction temperature.

As becomes clear from the gradual shift of the Zn3d peak maximum BE in Fig. S2 (b), a deconvolution of the Zn3d region into two distinct, fixed BE components for a single, uniform Zn(ox) surface species and purely bimetallic ZnCu is to some extent artificial. The BE shift can be attributed not only to oxidative segregation of a "single-phase" Zn(ox) species, but also to a gradual change of Cu-Zn and Zn-O(H) coordination chemistry. The peak fits of Figure 3b and 3d (main text) thus might underestimate the contribution of intermediate "interfacial" states of partially oxidised Zn, e.g. a wetting "2D" Zn-hydroxide layer with intrinsically higher $Cu^0$-coordination of Zn(ox), as compared to 3-dimensional ZnO islands with a minor contribution of Cu-ZnO interface species. At least, the fitting of the - with respect to both the 300K CuZn and 693K Zn(ox) spectra markedly broadened - peaks at 543K and 573K in Figure 3 (main paper) shows, that a contribution of bimetallic Zn is likely, which may be important for adsorption properties of the metallic surface (e.g. of formate species relevant to the reaction mechanism, as mentioned in the main text).

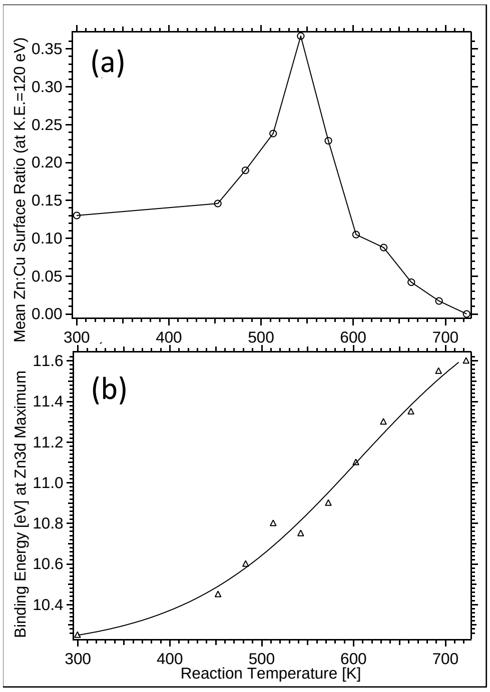


Fig. S2: (a) Mean Zn:Cu surface ratio derived from the Cu3d/Zn3d intensity ratio shown in Figure 3a and 3c (main text). (b) Change of binding energy at the Zn3d peak maximum with reaction temperature, as derived from the spectra of Figure 3 (main text).

**S3:** Probe-depth sensitive detection of the “interfacial Zn(ox)” state:

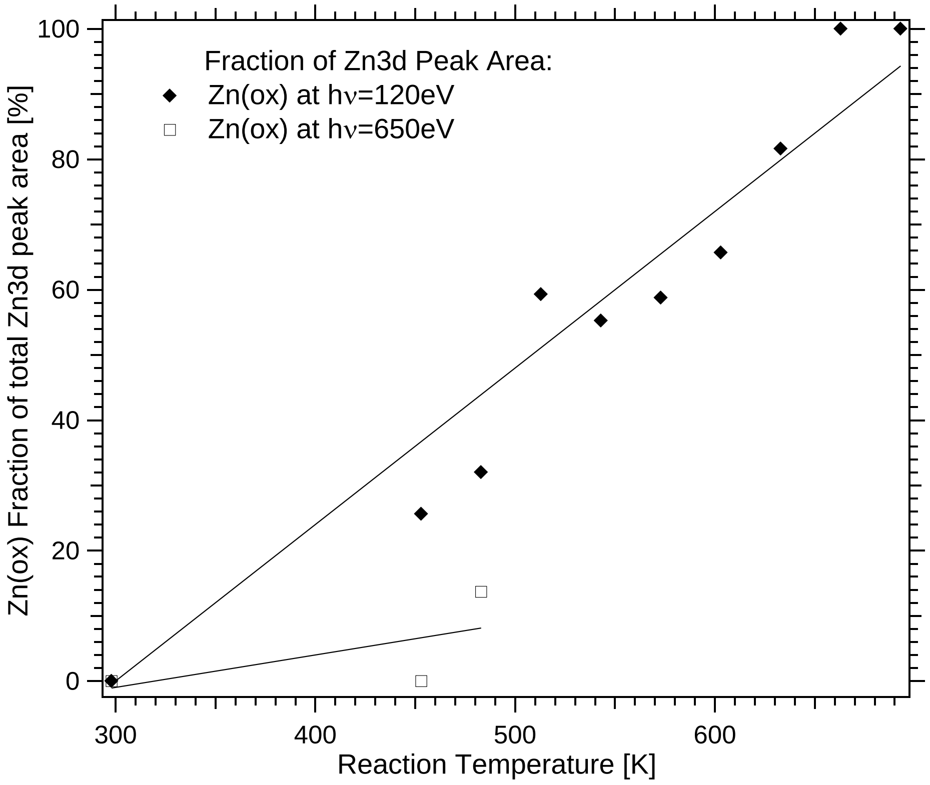


Fig. S3: Percent ratio of Zn(ox) with respect to the total peak area (Zn(bimetallic) + Zn(ox)), acquired in situ during MSR as a function of probe depth (photon energies: 130 and 650 eV) and reaction temperature. Data for 130 eV were calculated from the Zn3d peak fits of Figure 3, and analogous peak fits were performed for the 650eV spectra. The data indicate, that at least for temperatures up to 483K Zn(ox) is mainly a “flat” surface species, rather than 3-dimensional ZnO islands. By mistake, the Zn3d region was not recorded in the valence band scans at 650eV above 483K.

**S4:** Trends in the O1s core level binding energy and intensity recorded in situ during MSR, related to the Zn3d data of Figure 3. For assignment of typical O1s binding energies in oxidic and hydroxylated Zn and Cu species, see supporting literature [3-6]. The O1s intensity trend (shown in upper right Fig. S4) is closely related to the Zn3d trend of Figure 3c, exhibiting a maximum around 573 K. From ~600K to 723K not only the Zn3d-, but also the O1s-intensity drops strongly, meaning that most of the oxygen at the surface is associated with Zn rather than Cu (or to some extent with $C_1$-oxygenates, but missing intensity in the respective C1s BE range disfavours this option).

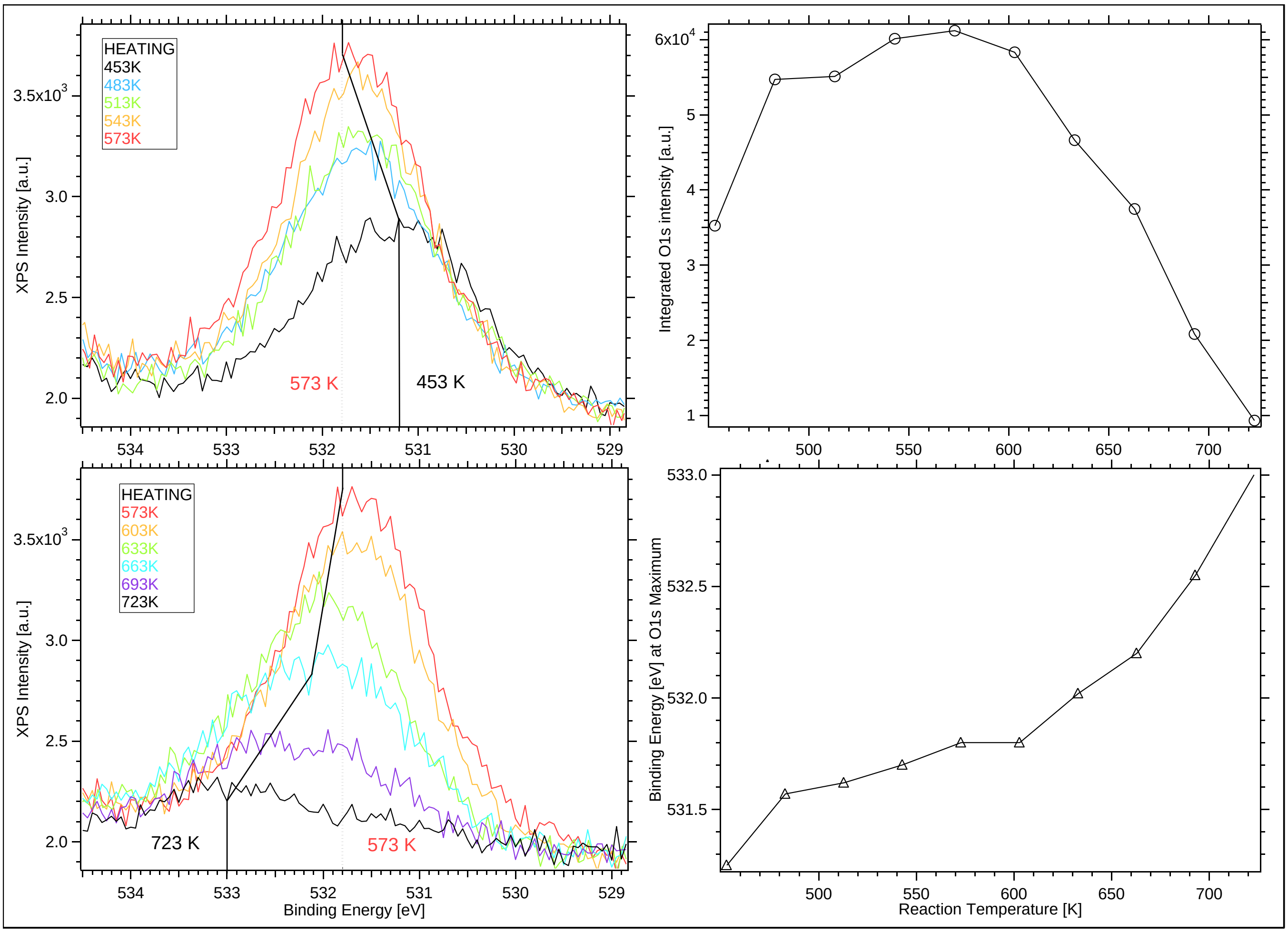


Fig. S4: AP-XPS spectra of the O1s signal acquired in situ during MSR on the initial CuZn~10:1 alloy, recorded with 650 eV photon energy. Upper left: O1s spectra from 453K to 573K. Lower left: O1s spectra from 573K to 723K. Upper right: Integrated O1s intensity as a function of reaction temperature. Lower right: Change of binding energy at the O1s peak maximum with reaction temperature.

**S5:** It is worthwhile to discuss why the ultra-clean, thermally annealed, structurally equilibrated copper foil used in our experiments represents a particularily unreactive state of Cu in (oxygen-free) MSR, and why several supported Zn-free Cu catalyst systems nevertheless exhibit high MSR- (and also methanol synthesis) activity.

Several potential Cu activators other than Zn have been discussed in the literature. Higher conversions of methanol were reported for $Cu/ZrO_2$ in comparison with $Cu/SiO_2$ prepared by impregnation methods [7,8]. A microemulsion-prepared $Cu/ZrO_2$ catalyst [9] with a Cu particle size of <10 nm reached activity levels identical to a commercial Cu/ZnO based catalyst, but at substantially reduced CO levels. In [9], oxidized $Cu^+$ species, being discussed to play an important role in MSR, were detected by XPS after reaction, in contrast to the Cu/ZnO reference catalyst studied in [9]. Decreased reducibility of $Cu^{2+}$ to $Cu^0$ due to the $Cu/ZrO_2$ contact, and thus higher abundance of residual oxygen in the Cu phase, was shown in [10] to enhance MSR activity.

Moreover, intrinsic MSR activity differences at the same nominal surface area of Cu could be verified, even on the same sample after different pretreatments [11]. The literature discussion is relating these intrinsic factors either to the *in situ* adjustment of the $Cu^0/Cu^+$ ratio at the catalyst's surface, or to the defect structure/disorder in metallic Cu, depending on differences in microstructure and preparation.

Variability of the oxidation state of Cu depending on the oxidation potential of the gas feed has been verified for oxidative MSR in [12]. $Cu^{2+}$ was shown to be MSR-inactive and $H_2$ production was only detected in the presence of $Cu^+/Cu^0$, with $Cu^+$ representing an intermediate in the reduction of $Cu^{2+}$ to $Cu^0$. In methanol partial oxidation on clean Cu foil, the dynamic near-surface Cu-O chemistry has been elucidated using AP-XPS [13]. Variations of the "in-situ" active state of Cu between sub-oxide and Cu(I)-oxide like species were found, with the most active and selective state to formaldehyde being a disordered $Cu^0$ surface modified by sub-surface oxygen of a composition near $Cu_{\sim 10}O$. The pure $Cu^0$ metal and bulk $Cu_2O$ phases were not sufficient to fully explain the catalytic performance of Cu. In related oxidative MSR studies of our group, strongly retarded activity on clean, unpromoted Cu foil (explained by slow $O_2$-induced self-activation) could be overcome by a preceding Cu activation treatment involving oxidation for 30 min in 200 mbar $O_2$ and reduction in 200 mbar methanol, both at 673K. In the related AP-XPS measurements, reversible formation of $O_{sub}$ (BE~529.1eV, besides $Cu_2O$ formation at BE~530.5 eV [13]) was observed to scale with $CO_2$ formation [14].

To evaluate potential catalytic effects of Cu surface roughening induced via preparation of the Cu:Zn~10:1 „pre-catalyst" state, we moreover performed control experiments with Zn-free Cu surfaces which were deliberately roughened and structurally disturbed by prolonged $Ar^+$-sputtering (6.0 x $10^{-5}$ mbar Ar, 2 keV, 1 µA sample current, 60 min) prior to the (otherwise identically performed) MSR rate measurements. One sputtered sample was used without subsequent thermal annealing, and another with thermal annealing at the Cu:Zn~10:1-compatible annealing temperature of 523 K. Interestingly, both the sputtered/non-tempered and sputtered/523K-tempered Cu surface showed indeed enhanced activity, but towards

formaldehyde (HCHO) instead of $CO_2$. From the rate data of Figure 2 for Cu:Zn~10:1 (main paper, upper panel) we can deduce a maximum HCHO formation rate of ~0.017 mbar/min, whereas the $CO_2$ formation shows a maximum of ~0.6 mbar/min. In contrast, the sputtered/non-tempered Cu yielded ~0.17 mbar/min HCHO but only ~0.06 mbar/min $CO_2$. The sputtered/523K-tempered sample yielded ~0.10 mbar/min HCHO and ~0.007 mbar/min $CO_2$. Finally, the clean 973K-tempered Cu (as already shown in Figure 2, lower panel) is even more $CO_2$-deactivated (only ~0.003 mbar/min) but also produced ~0.018 mbar/min HCHO (similar to Cu:Zn~10:1). The observed strong selectivity shift from $CO_2$ towards HCHO, as well as the (in comparison to Cu:Zn~10:1) much lower $CO_2$ formation rate observed both on sputtered/non-tempered and sputtered/523K-tempered Cu, supports our interpretation that Zn indeed represents a superior "water-activator" for optimization of total oxidation of HCHO towards $CO_2$.

In conclusion, we note that the view of potentially improved reaction channels on $Cu(Zn)^0$/Zn(ox) discussed in the main text does not contradict the occurrence of analogous, though differently weighted, processes on the above mentioned Zn-free supported Cu catalysts or on activated Cu surfaces. Differences in activity and selectivity may be correlated to the relative efficiency of water activation, the relative abundance of $O(H)_{ads}$ species, the relative ratio of decarboxylation vs. decarbonylation of formates etc.

Since the combined AP-XPS/ QMS analysis under MSR conditions shown in Figure 3 (main text) involves heating of the sample for several hours at temperatures well above 633K in a flow reaction system (i.e. continuously removing the $H_2$ formed), continuous oxidative activation of Cu during the experiment is conceivable. Progressive Zn loss can already explain the strong deviation from linearity in Fig. S5 above ~633K. Moreover, the rate does not drop to zero after (apparent) total loss of Zn at ~700K, likely due to simultaneous oxidative Cu-activation. In contrast, these effects are much more unlikely in the batch MSR reaction in setup 1, since the upper temperature limit of was as low as 623K and the gas phase exposure time at temperatures above 550K only ~30 min. Moreover, increasing surface reduction by the reaction product hydrogen, quickly replacing the reactants in the batch reactor, has to be considered.

Fig. S5 shows the Arrhenius plot of QMS intensity vs. inverse temperature for the in-situ measured hydrogen (mass 2) data obtained in setup 2. The activation barrier of ~98 kJ/mole in the temperature range below 633 K can be compared to $E_A$~93 kJ/mole derived from Fig S1.

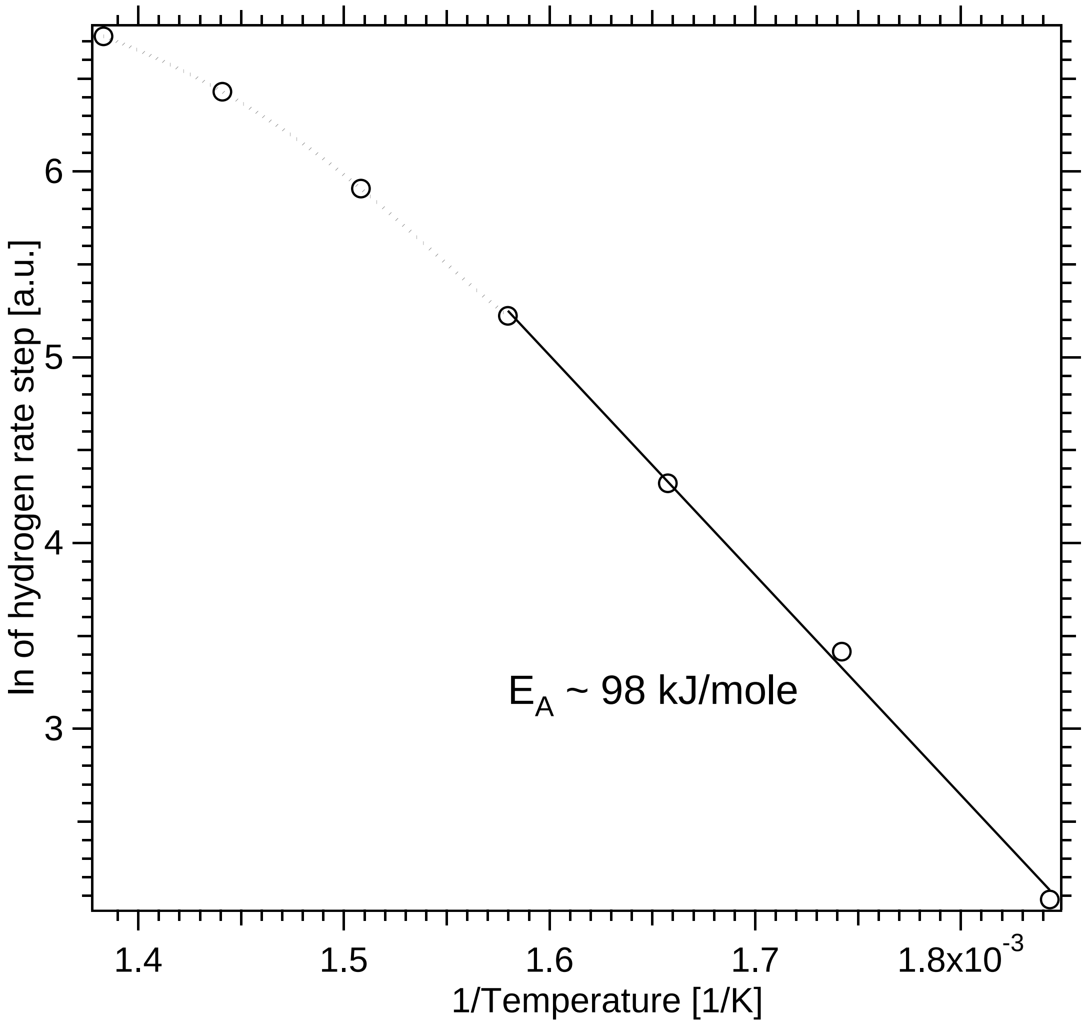


Fig. S5: Arrhenius analysis of $H_2$ formation rate equivalent QMS intensity measured at HZB/BESSY II between 543 K and 723 K.

**Supporting Literature:**

[1] W. Reichl, G. Rosina, G. Rupprechter, C. Zimmermann, K. Hayek, Rev. Sci. Instrum. 71(3) (2000) 1495.

[2] H. Bluhm, M. Hävecker, A. Knop-Gericke, E. Kleimenov, R. Schlögl, D. Teschner, V.I. Bukhtiyarov, D.F. Ogletree, M. Salmeron, J. Phys. Chem. B 108 (2004) 14340.

[3] M. Kunat, St. Gil Girol, U. Burghaus, Ch. Wöll, J. Phys. Chem. B 107 (2003) 14350.

[4] G. Ghiotti, A. Chiorino, F. Boccuzzi, Surf. Sci. 287/288 (1993) 228.

[5] I. Platzman, R. Brener, H. Haick, R. Tannenbaum, J. Phys. Chem. C 2008, 112, 1101.

[6] R.A. Zarate, F. Hevia, S. Fuentes, V.M. Fuenzalida, A. Zúñiga, J. Solid State Chem. 180 (4) (2007) 1464.

[7] H. Kobayashi, N. Takezawa, M. Shimokawabe, K. Takahashi, Stud. Surf. Sci. Catal. 16 (1983) 697.

[8] N. Takezawa, M. Shimokawabe, H. Hiramatsu, H. Sugiura, T. Asakawa, H. Kobayashi, React. Kinet. Catal. Lett. 33 (1987) 191.

[9] I. Ritzkopf, S. Vukojevic, C. Weidenthaler, J.D. Grunwaldt, F. Schüth, Appl. Catal. A 302 (2006) 215.

[10] A. Szizybalski, F. Girgsdies, A. Rabis, Y. Wang, M. Niederberger, T. Ressler, J. Catal. 233 (2005) 297.

[11] M.M. Günther, T. Ressler, R.E. Jentoft, B. Bems, J. Catal. 203 (2001) 133.

[12] T.L. Reitz, P.L. Lee, K.F. Czaplewski, J.C. Lang, K.E. Popp, H.H. Kung, J. Catal. 199 (2001) 193.

[13] A. Knop-Gericke, M. Hävecker, T. Schedel-Niedrig, R. Schlögl, Top. Catal. 15 (2001) 27.

[14] C. Rameshan, Dissertation, University of Innsbruck, 2011.

**Experimentelles**

Als Referenzkatalysator und Substrat für CuZn Oberflächenlegierungen wurde ultra-reine Kupferfolie (Goodfellow, Reinheit 99,999%; 0,10 mm Dicke, 3,5 $cm^2$ Fläche) ohne weitere Aktivierung verwendet. Zur Präparation und Charakterisierung einer sauberen Oberfläche wurden mehrere Zyklen von Argonsputtern mit nachfolgendem thermischen Ausheilen (6,0x$10^{-5}$ mbar Ar, 2 keV, 1 µA Probenstrom, T = 973 K) durchgeführt, bis die XPS und AES Spektren keine Spuren von Verunreinigungen aufwiesen. Um die Oberflächenlegierung herzustellen, wurden fünf oder zwölf Monolagen metallisches Zn auf die saubere Kupferoberfläche aufgebracht, und anschließend auf Temperaturen zwischen 300 und 653 K geheizt. Die Messing-Referenzproben mit Zn-Anteilen von 10, 15 oder 37% (Gewicht) stammten von Goodfellow (CuZn37) und Schlenk Metallfolien (CuZn10 und CuZn15).

Die Messungen wurden an zwei verschiedenen Apparaturen durchgeführt:

*Setup 1: UHV-Oberflächenpräparations- und Analysen-Kammer mit angeschlossenem Realdruck-Reaktor*

Das UHV-System ist an einen Zirkulationsreaktor aus Glas angeschlossen (nähere Beschreibung in [1]), welcher für quantitative katalytische/kinetische Untersuchungen von polykristallinen Folien bei bis zu 1 bar entworfen wurde. Der Nachweis der Produkte erfolgte mittels Online-MS (HP GC-MS System G1800A) durch eine Kapillare und/oder mittels konventionellem (Injektions-)GC-MS. Die MS-Signale von $CO_2$, CO, $H_2$ und $CH_2O$ wurden extern kalibriert und um die Fragmentierung korrigiert. So konnte auch die stöchiometrische Reformierung von Methanol zu $CO_2/H_2$=1/3 verifiziert werden. Die Proben wurden weiters mittels eines XPS/AES/LEIS Spektrometers (Thermo Electron Alpha 110) und einer Twin Mg/Al anode X-ray gun (XR 50, SPECS) untersucht. Die Deposition von Zink aus einer selbstgebauten Knudsenzelle, welche mit Zinkschrot gefüllt ist (Sigma-Aldrich, 99,999%) wurde mittels Schwingquarz kontrolliert. Alle MSR Messungen erfolgten unter Verwendung von entgasten Methanol/Wasser Mischungen bei einer Zusammensetzung von 1:10 in der flüssigen Phase, was bei 300 K einem Partialdruckverhältnis von Methanol:Wasser=1:2 entspricht. Der Reaktor wurde mit einer konstanten Heizrate von circa 9 K/min auf die Maximaltemperatur von 623 K gefahren und dort für ~25 min isotherm gehalten. Die Reaktionsraten wurden durch Differentiation der Produkt-Partialdrücke gegen die Zeit errechnet und sind in Veränderungen des Partialdrucks pro Minute (mbar/min) angegeben

*Setup 2: AP-XPS Spektrometer für in-situ Charakterisierung des Katalysators bei realitätsnahen Reaktionsbedingungen*

Die Vakuumkammer, welche am HZB/BESSY II (an der Beamline ISISS-PGM) betrieben wird, erlaubte es uns, *in situ* Photoelektronenspektroskopie bei Drücken von bis zu 1 mbar Druck aufzunehmen [2]. Die Cu-Folie, beziehungsweise die CuZn Proben, wurden innerhalb der Analysekammer ungefähr 2 mm vor einer 1 mm Blende positioniert, welche den Eingang eines differentiell gepumpten, elektrostatischen Linsensystems darstellt, das Gasmoleküle von den auf den SPECS Halbkugelanalysator gerichteten Photoelektronen trennt. Bindungsenergien sind auf die Fermikante bezogen, welche nach jeder Messung aufgezeichnet wurde.

Die elementare Zusammensetzung in Atomprozent wurde als Funktion der Analysentiefe aus den Cu3p und Zn3p XPS-Signalen abgeleitet, wobei die Photonenenergie durch Subtraktion von ~90 eV für Zn3p und ~75 eV für Cu3p in die kinetische Energie umgerechnet werden kann. Die Wirkungsquerschnitte zur Berechnung des Cu:Zn Verhältnisses wurden den Daten von http://ulisse.elettra.trieste.it/services/elements/WebElements.html entnommen. Da die Cu3p und Zn3p Bereiche mit derselben Photonenenergie und in einem Schritt aufgezeichnet wurden, ist eine Korrektur um den Photonenfluss nicht notwendig.

Die Temperatur wurde mittels eines K-Typ Ni/NiCr Thermoelements gemessen, welches auf eine Seite der Probe punktgeschweißt wurde. Temperaturprogrammiertes Heizen erfolgte mittels eines IR-Lasers von der Rückseite. Die Reinigung des Cu-Substrats erfolgte durch wiederholtes Sputtern mit $Ar^+$ bei Raumtemperatur, gefolgt von thermischem Ausheilen bei 950 K im UHV. Anschließend wurde die Probe zuerst in $O_2$ und dann im UHV für 60 s auf 950 K geheizt.

Die CuZn Oberflächenlegierungen wurden im *Setup 1* hergestellt und dann zum HZB/BESSY II transportiert. Da sie zwischen ihrer Herstellung und der spektroskopischen Analyse Umgebungsbedingungen ausgesetzt waren, zeigten sich Zeichen von oxidativer Zn Segregation zur Oberfläche, speziell bei niederen kinetischen Energien der Photoelektronen. Auf den lediglich niedrig getemperten Proben (≤400 K) war diese Segregation ausgeprägt, während die auf 523 K erhitzten Proben unter Umgebungsbedingungen stabiler waren, also auch in den obersten Lagen nur vernachlässigbare Anreicherung von oxidiertem Zn zeigten.

Die AP-XPS Untersuchung des Modellkatalysators unter MSR Bedingungen wurde durch gleichzeitige Quadrupol-Massenspektrometrie (QMS)-basierte Messung der Produkt- und Reaktanden-Partialdrücke ergänzt. Anders als bei den wesentlich genaueren Ratenmessungen im Zirkulationsreaktor von *Setup 1*, wurde die Analyse der Veränderung der Zusammensetzung der Gasphase am HZB/BESSY II in einem Strömungsreaktor gemessen. Dabei waren auch Temperaturen jenseits von 623 K zugänglich.

## Zusatzinformationen

**S1:** Abschätzung der Aktivierungsenergie für die $CO_2$-Bildung auf der anfänglichen CuZn~10:1 Probe im Zirkulationsreaktor von Setup 1, basierend auf den $CO_2$-Bildungsraten von Abbildung 2 (siehe Haupttext). Um die Abhängigkeit der Rate von Veränderungen der Reaktanden- und Produktdrücke möglichst zu entkoppeln, wurde die Analyse auf die Anfangsrate zwischen 530 und 550 K beschränkt, wo noch kaum Reaktandenverbrauch oder Produktbildung stattfinden.

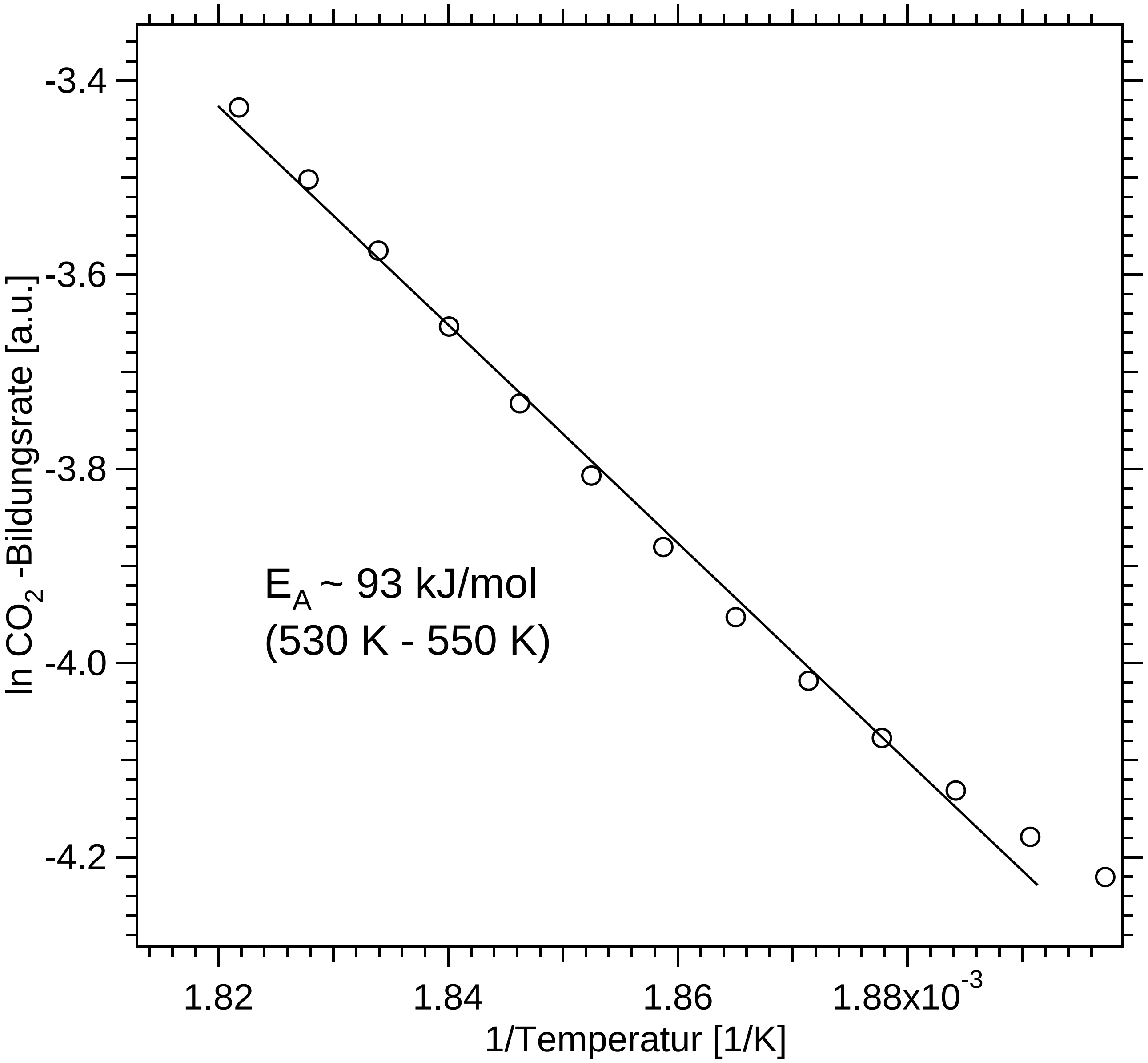


Abbildung S1: Arrhenius-Analyse der im Strömungsreaktor gemessenen $CO_2$ Bildungsrate, gemessen in *Setup 1*, zwischen 530 und 550 K (vergleiche Abbildung 2 im Haupttext).

**S2:** Die Zusatzinformation zu Abbildung 3 (Haupttext) ist in Abbildung S2 dargestellt. (a) Quantifizierung des mittleren Zn/Cu-Verhältnisses unter Zugrundelegung der Cu3d/Zn3d Intensitäten. (b) Änderung der Bindungsenergie des Zn3d Peakmaximums mit der Reaktionstemperatur. Die kontinuierliche Verschiebung des Zn3d Peakmaximums in Abbildung S2(b) zeigt, dass eine Zerlegung des Zn3d Bereichs in zwei getrennte, fixe Bindungsenergiebeiträge für eine einzige Zn(ox) Oberflächenspezies und eine rein metallische ZnCu Oberflächenspezies bis zu einem gewissen Grad artifiziell ist. Die Änderung der Bindungsenergie kann nicht nur einer oxidativen Segregation einer einzigen Zn(ox) Spezies zugeschrieben werden, sondern auch einer kontinuierlichen Änderung der Cu-Zn- und Zn-O(H)-Koordinationschemie. Die Fits in den Abbildungen 3b und 3d im Haupttext unterschätzen daher wohl den Beitrag solcher Zwischenzustände von partiell oxidiertem Zn, wie zum Beispiel einer benetzenden „2D-Zn-Hydroxid"-Schicht mit intrinsisch höherer $Cu^0$ Koordination von Zn(ox), im Vergleich zu dreidimensionalen ZnO Inseln mit einem nur geringen Beitrag von Cu-ZnO-Grenzflächenspezies. Zumindest zeigt ein Vergleich deutlich verbreiterte Peaks bei 543 und 573 K relativ zu den Spektren von rein bimetallischem CuZn bei 300 K beziehungsweise von Zn(ox) bei 693 K (vgl. Abbildung 3, Haupttext), sodass ein Intensitätsbeitrag von metallischem Zn wahrscheinlich ist. Dies könnte wiederum für das Adsorptionsverhalten (zum Beispiel der für den Reaktionsmechanismus relevanten Formiatspezies, siehe Haupttext) bedeutend sein.

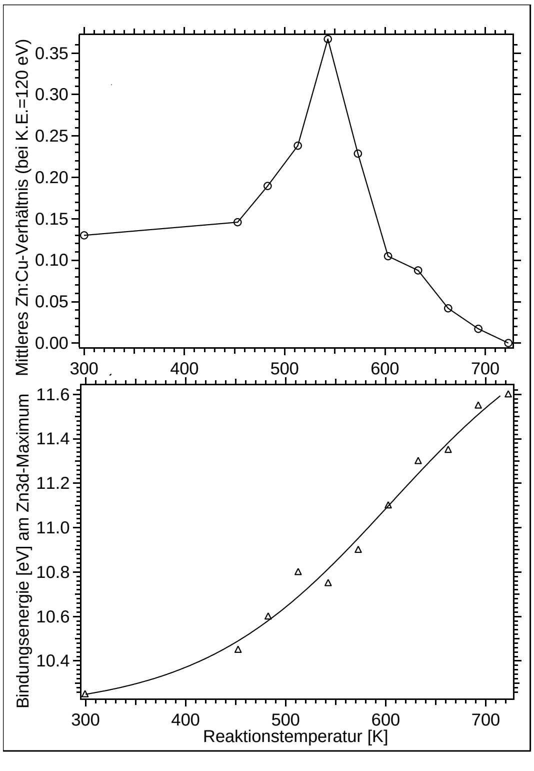


Abbildung S2: (a) Mittleres Zn/Cu Oberflächenverhältnis, abgeleitet vom Cu3d/Zn3d Intensitätsverhältnis aus Abbildung 3a und 3c (Haupttext). (b) Änderung der Bindungsenergie der Zn3d-Komponente mit der Reaktionstemperatur, abgeleitet aus den Spektren in Abbildung 3 (Haupttext).

**S3:** Zn(ox) Gehalt bei verschiedenen Informationstiefen:

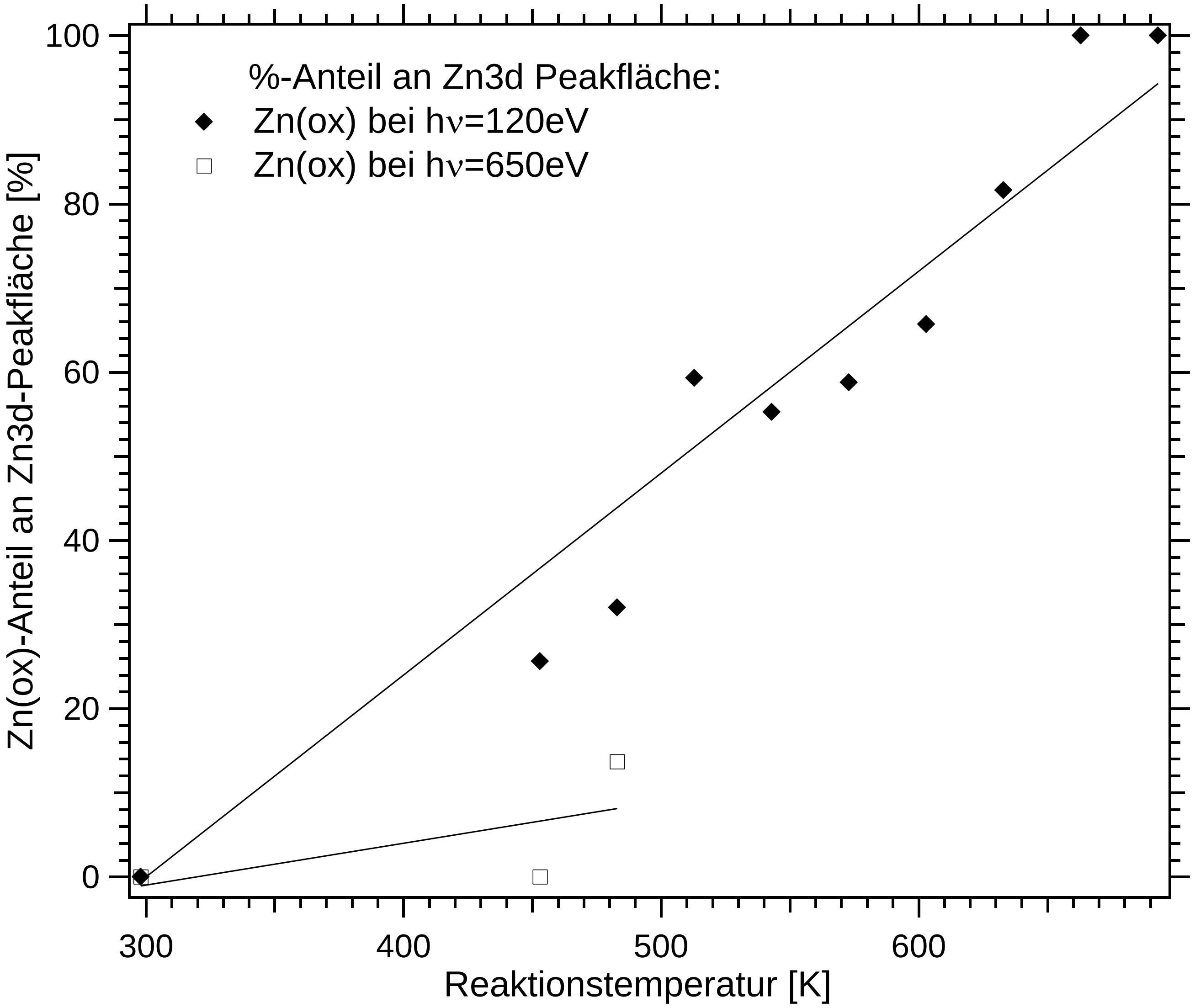


Abbildung S3: Anteil an Zn(ox) bezogen auf die gesamte Zn3d Peakfläche (Zn(bimetallisch)+Zn(ox)), aufgenommen in-situ während MSR bei verschiedenen Informationstiefen (Photonenenergie 130 eV beziehungsweise 650 eV) als Funktion der Reaktionstemperatur. Die Daten für 130 eV sind den Peakfits von Abbildung 3 (Haupttext) entnommen. Analoge Peak-Zerlegungen wurden auch für die 650 eV Spektren durchgeführt. Die Daten zeigen, dass Zn(ox) - zumindest bis 483 K - eher eine „flache“ Oberflächenschicht bildet und nicht dreidimensionale ZnO Inseln. Durch ein Versehen wurden Spektren mit 650 eV für Temperaturen oberhalb von 483 K nicht aufgezeichnet.

**S4:** Trends in der Bindungsenergie und Intensität des O1s Core Levels, aufgenommen während MSR, zum Vergleich mit den Zn3d Daten in Abbildung 3 (Haupttext). Für die Zuweisung typischer O1s Bindungsenergien in oxidischen und hydroxylierten Zn und Cu Spezies, siehe Zusatzliteratur [3-6]. Der O1s-Intensitätstrend (Abbildung S4, rechts oben), welcher ein Maximum bei ungefähr 573 K aufweist, ist eng mit dem Zn3d-Verlauf in Abbildung S2a verknüpft. Zwischen 600 und 723 K sinkt nicht nur das Zn3d Signal, sondern auch die O1s-Intensität stark ab, da der Großteil des Sauerstoffs an der Oberfläche eher mit Zn als mit Cu verbunden ist (oder in gewissem Ausmaß mit $C_1$-Oxygenaten, aber die fehlenden Intensitäten im Bereich der betreffenden C1s-Bindungsenergien sprechen gegen diese Möglichkeit).

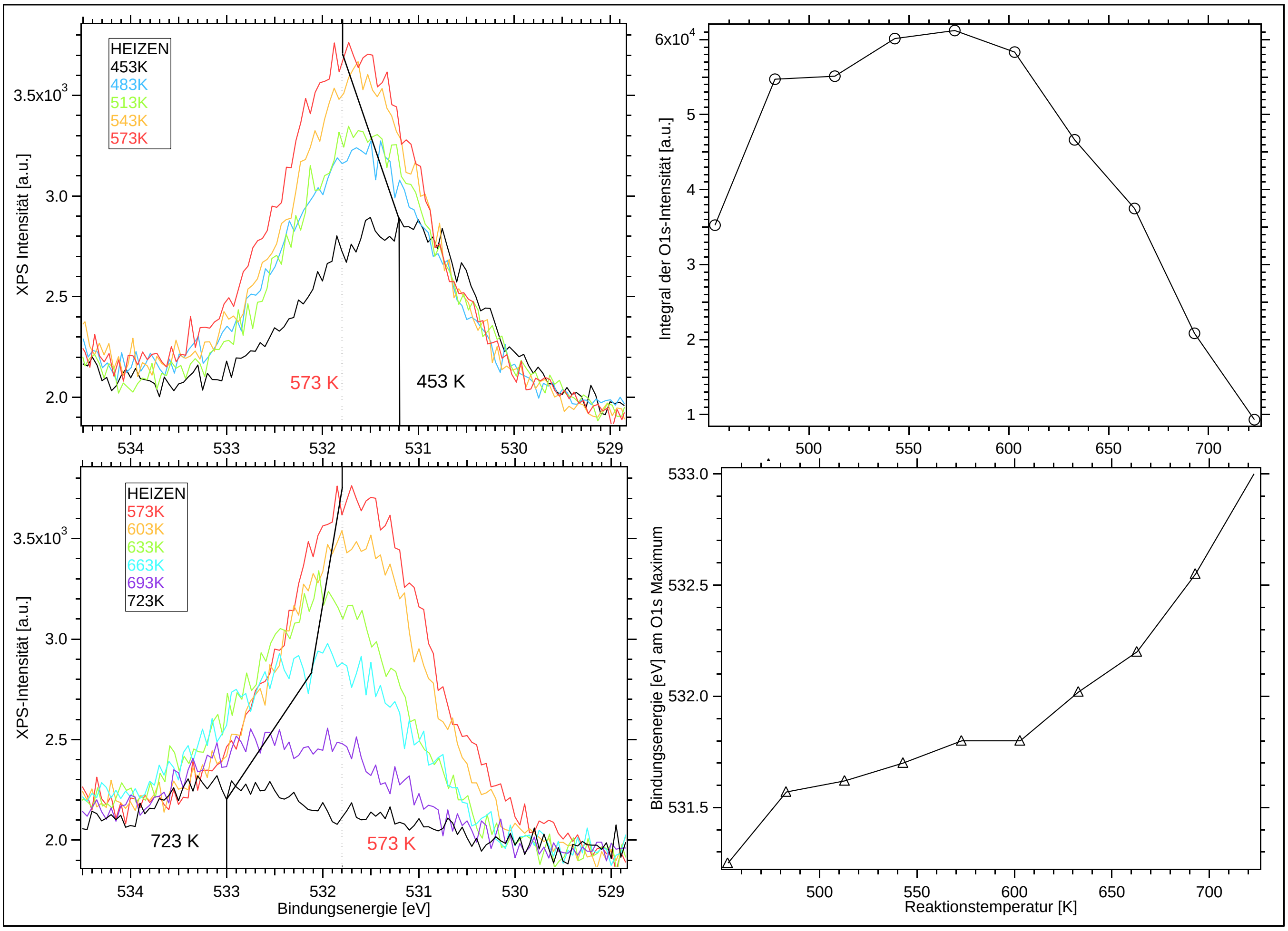


Abbildung S4: AP-XPS Spektren des O1s Signals während MSR auf der anfänglichen CuZn~10:1 Legierung, aufgenommen mit 650 eV Photonenenergie. Oben links: O1s-Spektren von 453 bis 573 K. Unten links: O1s-Spektren von 573 bis 723 K. Oben rechts: Integrierte O1s Intensität als Funktion der Reaktionstemperatur. Unten rechts: Veränderung der Bindungsenergie des O1s-Peakmaximums mit der Reaktionstemperatur.

**S5:** Interessanterweise stellt die hochreine, thermisch ausgeheilte, strukturell relaxierte Kupferfolie in unseren Experimenten einen besonders unreaktiven Zustand von Kupfer in (sauerstofffreier) MSR dar, während viele geträgerte zinkfreie Kupferkatalysatoren eine hohe MSR- (und auch Methanolsynthese-) Aktivität zeigen.

In der Literatur wurden verschiedene andere Cu Aktivatoren außer Zn untersucht. So wurde für einen imprägnierten $Cu/ZrO_2$-Katalysator ein im Vergleich zu $Cu/SiO_2$ erhöhter Umsatz von Methanol gefunden [7,8]. Ein $Cu/ZrO_2$ Katalysator, der aus einer Mikroemulsion hergestellt wurde und eine Partikelgröße von weniger als 10 nm aufwies [9], konnte die gleichen Aktivitäten erreichen wie ein kommerzieller Cu/ZnO-basierter Katalysator, aber bei wesentlich niedrigerer CO Produktion. In Referenz [9] wurden mittels XPS nach der Reaktion oxidierte $Cu^+$ Spezies nachgewiesen, deren Bedeutung für die MSR diskutiert wird, weil sie beim in [9] untersuchten Cu/ZnO-Referenz-Katalysator fehlten. Es konnte in [10] gezeigt werden, dass die durch den $Cu/ZrO_2$ Kontakt verringerte Reduzierbarkeit von $Cu^{2+}$ zu $Cu^0$, und - damit einhergehend - eine größere Menge an verbleibendem Sauerstoff in der Kupferphase die MSR-Aktivität positiv beeinflussen.

Weiters konnten auch auf nominell gleichen Flächen von Kupfer variable MSR Aktivitäten gemessen werden, wenn die Probe verschiedenen Vorbehandlungen unterworfen wurde [11]. In der Literatur werden diese intrinsischen Unterschiede entweder auf die *in situ* Einstellung des $Cu^0/Cu^+$ Verhältnisses oder auf Defekte und Unordnungsphänomene im metallischen Kupfer in Abhängigkeit von Mikrostruktur und Präparation zurückgeführt.

Eine Veränderung des Oxidationszustandes von Cu in Abhängigkeit vom Oxidationspotential des verwendeten Gases konnte bereits für oxidative MSR gezeigt werden [12]. $Cu^{2+}$ ist MSR-inaktiv, eine $H_2$-Entwicklung wurde nur in Anwesenheit von $Cu^+/Cu^0$ festgestellt, wobei $Cu^+$ einen Zwischenzustand in der Reduktion von $Cu^{2+}$ zu $Cu^0$ darstellt. Die oberflächennahe Cu-O Chemie bei der Partialoxidation von Methanol auf reiner Kupferfolie wurde mittels AP-XPS aufgeklärt [13]. Es wurden „in situ“ Veränderungen des aktiven Zustandes von Cu zwischen sub-oxidisch und Cu(I)-oxid gefunden, wobei der aktivste und selektivste Zustand für die Bildung von Formaldehyd eine ungeordnete $Cu^0$ Oberfläche ist, die knapp unter der Oberfläche eine Zusammensetzung von etwa $Cu_{\sim 10}O$ aufweist. Reines $Cu^0$ Metall und Bulk-$Cu_2O$-Phasen konnten die katalytischen Eigenschaften von Cu nicht befriedigend erklären. In von unserer Gruppe durchgeführten Studien zu oxidativer MSR konnte gezeigt werden, dass eine stark verzögerte Aktivität auf sauberer Cu Folie (erklärbar durch langsame, sauerstoffinduzierte Selbstaktivierung) durch einen vorhergehenden Aktivierungsschritt (Oxidation für 30 min in 200 mbar $O_2$, dann Reduktion in 200 mbar Methanol, beides bei 673 K) überwunden werden kann. In den dazugehörigen AP-XPS Messungen konnte die reversible Bildung von $O_{sub}$ (BE~529,1 eV, $Cu_2O$ Bildung ~530,5 eV [13]) mit der Bildung von $CO_2$ korreliert werden [14].

Um den möglichen Einfluss einer Aufrauung der Cu Oberfläche durch die Präparation des CuZn~10:1 Präkatalysators auf das katalytische Verhalten aufzuklären, wurden MSR-Experimente mit zinkfreien Kupferproben durchgeführt, welche vorher absichtlich durch $Ar^+$-Sputtern (6.0 x $10^{-5}$ mbar Ar, 2 keV, 1 µA Probenstrom, 60 min) aufgeraut wurden. Eine gesputterte Probe wurde nachträglich auf die zur CuZn~10:1 Legierungsbildung erforderliche Temperatur von 523 K geheizt, eine andere

wurde thermisch unbehandelt belassen. Beide Proben zeigten interessanterweise eine höhere Aktivität, allerdings für die Produktion von Formaldehyd (HCHO) anstatt von $CO_2$. Die CuZn~10:1 Legierung aus Abbildung 2 (Haupttext, oben) zeigt eine HCHO Bildungsrate von circa 0,017 mbar/min, während die $CO_2$-Bildung ein Maximum von ungefähr 0,6 mbar/min aufweist. Die gesputterte, aber nicht geheizte Cu-Probe zeigte Bildungsraten von 0,17 mbar/min für HCHO, aber nur noch 0,06 mbar/min für $CO_2$. Die gesputterte und auf 524 K geheizte Probe wies Bildungsraten von 0,10 mbar/min für HCHO und circa 0,007 mbar/min für $CO_2$ auf. Die reine, auf 973 K geheizte Kupferprobe schließlich (Abbildung 2 im Haupttext, unten) ist bezüglich der Produktion von $CO_2$ sogar noch weniger aktiv (nur mehr ~0,003 mbar/min), produziert aber trotzdem 0,018 mbar/min HCHO, so wie die CuZn~10:1 Legierung. Der beobachtete Selektivitätswechsel von $CO_2$ in Richtung HCHO, sowie die im Vergleich zur CuZn~10:1 Legierung viel niedrigere $CO_2$ Bildungsrate auf den beiden gesputterten Proben unterstützen unsere Interpretation, dass Zn tatsächlich einen überragenden „Wasseraktivator" für die Optimierung der Totaloxidation von HCHO zu $CO_2$ darstellt.

Abschließend sei festgestellt, dass die im Haupttext diskutierten potentiell verbesserten Reaktionskanäle auf $Cu(Zn)^0$/Zn(ox) nicht dem Auftreten analoger, allerdings anders gewichteter Prozesse, auf den oben genannten zinkfreien geträgerten Kupferkatalysatoren oder auf anderweitig aktivierten Kupferoberflächen widersprechen. Die Unterschiede in Aktivität und Selektivität könnten mit der relativen Effizienz der Wasseraktivierung, der relativen Häufigkeit von $O(H)_{ads}$ Spezies, den relativen Raten der Decarboxylierung im Vergleich zur Decarbonylierung von Formiaten und anderen Faktoren zusammenhängen.

Da die kombinierte AP-XPS/QMS Analyse unter MSR Bedingungen in Abbildung 3 (Haupttext) ein Heizen der Probe von mehreren Stunden bei Temperaturen über 633 K in Reaktionsatmosphäre erfordert, ist eine oxidative Aktivierung von Cu im Strömungsreaktorsystem bemerkbar (da das gebildete $H_2$ ständig abgeführt wird). Der fortschreitende Verlust an Zn kann die starken Abweichungen von der Linearität in Abbildung S5 oberhalb von 633 K erklären. Außerdem fällt die Rate selbst nach dem (sichtbaren) Verschwinden allen Zinks bei ~ 700 K nicht auf Null, wahrscheinlich wegen der erwähnten oxidativen Kupferaktivierung. Im Zirkulationsreaktor von *Setup 1* sind diese Effekte hingegen viel unwahrscheinlicher, da das obere Temperaturlimit bereits bei 623 K erreicht ist und die Probe der Gasphase oberhalb von 550 K nur ungefähr 30 min lang ausgesetzt ist. Außerdem muss eine verstärkte Oberflächenreduktion durch das Reaktionsprodukt Wasserstoff, welches die Reaktanden im Reaktor alsbald ersetzt, berücksichtigt werden.

Abbildung S5 zeigt die Arrheniusauftragung der QMS-Intensität gegen die inverse Temperatur für die in situ gemessenen Wasserstoffdaten (Masse 2), welche in *Setup 2* gewonnen wurden. Die Aktivierungsenergie von ~98 kJ/mol im Temperaturbereich unter 633 K ist mit $E_A$~93 kJ/mol aus Abbildung S1 vergleichbar.

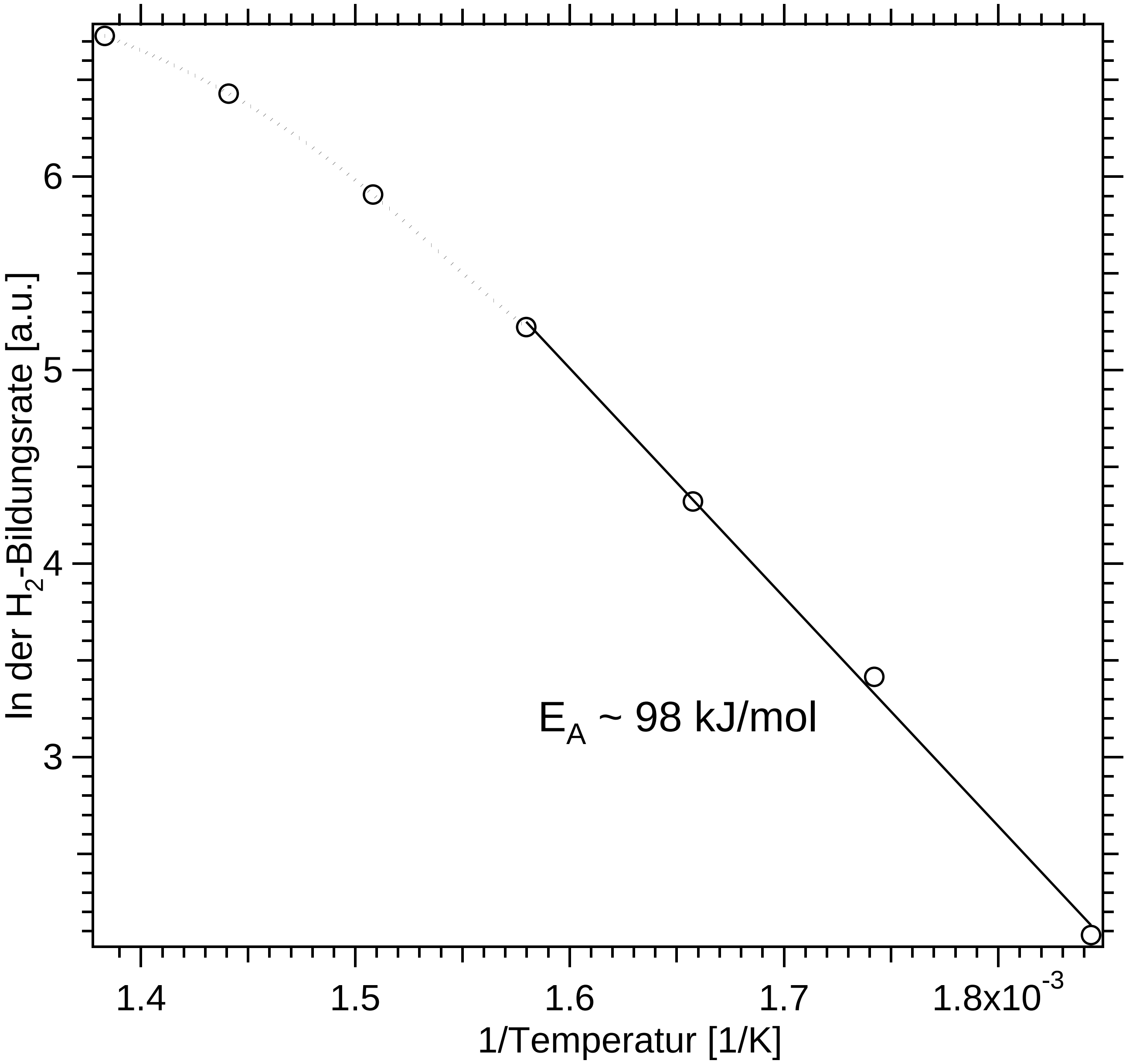


Abbildung S5: Arrhenius Auftragung des der $H_2$ Bildungsrate äquivalenten QMS Signals, gemessen am HZB/BESSY II zwischen 543 und 723 K.

**Zusatzliteratur:**

[1] W. Reichl, G. Rosina, G. Rupprechter, C. Zimmermann, K. Hayek, Rev. Sci. Instrum. 71(3) (2000) 1495.

[2] H. Bluhm, M. Hävecker, A. Knop-Gericke, E. Kleimenov, R. Schlögl, D. Teschner, V.I. Bukhtiyarov, D.F. Ogletree, M. Salmeron, J. Phys. Chem. B 108 (2004) 14340.

[3] M. Kunat, St. Gil Girol, U. Burghaus, Ch. Wöll, J. Phys. Chem. B 107 (2003) 14350.

[4] G. Ghiotti, A. Chiorino, F. Boccuzzi, Surf. Sci. 287/288 (1993) 228.

[5] I. Platzman, R. Brener, H. Haick, R. Tannenbaum, J. Phys. Chem. C 2008, 112, 1101.

[6] R.A. Zarate, F. Hevia, S. Fuentes, V.M. Fuenzalida, A. Zúñiga, J. Solid State Chem. 180 (4) (2007) 1464.

[7] H. Kobayashi, N. Takezawa, M. Shimokawabe, K. Takahashi, Stud. Surf. Sci. Catal. 16 (1983) 697.

[8] N. Takezawa, M. Shimokawabe, H. Hiramatsu, H. Sugiura, T. Asakawa, H. Kobayashi, React. Kinet. Catal. Lett. 33 (1987) 191.

[9] I. Ritzkopf, S. Vukojevic, C. Weidenthaler, J.D. Grunwaldt, F. Schüth, Appl. Catal. A 302 (2006) 215.

[10] A. Szizybalski, F. Girgsdies, A. Rabis, Y. Wang, M. Niederberger, T. Ressler, J. Catal. 233 (2005) 297.

[11] M.M. Günther, T. Ressler, R.E. Jentoft, B. Bems, J. Catal. 203 (2001) 133.

[12] T.L. Reitz, P.L. Lee, K.F. Czaplewski, J.C. Lang, K.E. Popp, H.H. Kung, J. Catal. 199 (2001) 193.

[13] A. Knop-Gericke, M. Hävecker, T. Schedel-Niedrig, R. Schlögl, Top. Catal. 15 (2001) 27.

[14] C. Rameshan, Dissertation, University of Innsbruck, 2011.
